# Influence of Sintering and Heat Treatment on the Microstructure, Mechanical Properties, and Tribological Performance of AlTiN-Coated PM M42 High-Speed Steel

**DOI:** 10.3390/ma19081667

**Published:** 2026-04-21

**Authors:** Zijun Qi, Yi Chen, Ji Li, Yongde Huang, Qian Wang, Qi Wei, Xiaofeng Yang, Qiang Liu

**Affiliations:** 1 School of Material Science and Engineering, Nanchang Hangkong University, Nanchang 330063, China; 2303082500009@stu.nchu.edu.cn (Z.Q.); 70793@nchu.edu.cn (Y.C.); 70844@nchu.edu.cn (Q.L.); 2 University of Science and Technology of China, Hefei 230026, China; lj17730835968@mail.ustc.edu.cn; 3 Key Laboratory of Rare Earths, Ganjiang Innovation Academy, Chinese Academy of Sciences, Ganzhou 341119, China; xiaofengyang@mail.ustc.edu.cn; 4 School of Intelligent Manufacturing (Aerospace Engineering), Taizhou University, Taizhou 318000, China; huangydhm@tzc.edu.cn

**Keywords:** M42 high-speed steel, AlTiN coating, carbide phase evolution, friction and wear, mechanical properties, wear rate

## Abstract

**Highlights:**

- Elevating the sintering temperature promoted densification of M42 high-speed steel, thereby concurrently improving both its hardness and bending strength (σ).
- Microstructural evolution during austenitizing, consistent with a possible phase transformation from M_2_C to MC and M_6_C, leads to a trade-off between strength and toughness. The hardness exhibits an initial increase followed by a subsequent decrease, reaching a peak value of 868 HV at 60 min, while the bending strength decreases monotonically.
- The microstructural evolution of the matrix carbides appears to enhance the wear resistance of the AlTiN coating by synergistically regulating the microstructure and mechanical properties (Vickers hardness, bending strength σ, and interfacial bonding strength L_C_) of the matrix.
- The AlTiN coating deposited on the M42 high-speed steel substrate sintered at 1190 °C and austenitized for 120 min exhibits the lowest wear rate (2.38 × 10^−6^ mm^3^·N^−1^·m^−1^), demonstrating superior wear resistance.

**Abstract:**

Preparing a highly wear-resistant AlTiN coating on a powder metallurgy (PM) M42 high-speed steel substrate is a key strategy to enhance tool performance and meet the demands of efficient machining. This study adopted a process route comprising substrate preparation, heat treatment regulation, and arc-PVD deposition of AlTiN coatings to systematically investigate the influence of sintering temperature (1130, 1160, and 1190 °C) and austenitizing time (1150 °C for 0, 15, 60, and 120 min) on the microstructure and mechanical properties of the substrate, as well as on the tribological performance of the AlTiN coatings. The results indicate that elevating the sintering temperature promotes densification of the matrix, with Vickers hardness increasing from 366 HV to 462 HV and bending strength (σ) increasing from 1064 MPa to 1310 MPa. The predominant carbide phases identified are MC, M_2_C, and M_6_C. During austenitizing, microstructural changes consistent with a progressive transformation from M_2_C to MC and M_6_C carbides were indicated by SEM and XRD analyses. Precipitation strengthening was most evident after 60 min, with hardness reaching 868 HV. In contrast, bending strength (σ) exhibited a progressive decline with increasing austenitizing time, decreasing from 1310 MPa to 1015 MPa after 120 min, illustrating a clear trade-off between hardness and toughness. The wear behavior of the coating is governed synergistically by substrate hardness, bending strength (σ), coating–substrate interfacial adhesion strength (L_C_), and carbide phase transformation. Elevated substrate hardness enhances anti-wear performance; bending strength influences crack propagation and spallation tendency; and L_C_ determines the efficiency of interfacial load transfer. The carbide phase evolution appears to modulate the coating’s wear behavior by regulating both the microstructure and mechanical properties of the substrate. Among the six sample conditions evaluated, the A3 sample (sintered at 1190 °C and austenitized for 120 min) exhibited the lowest wear rate (2.38 × 10^−6^ mm^3^·N^−1^·m^−1^), demonstrating superior wear resistance. These findings provide a reference for process optimization and rational design of M42/AlTiN composite coating systems.

## 1. Introduction

With the continuous improvement of processing efficiency and accuracy requirements in modern manufacturing, the comprehensive service performance of cutting tool materials has become a key bottleneck restricting the sustainable development of advanced cutting technologies [1]. High-speed steel (HSS), particularly M42 HSS prepared by powder metallurgy (PM), is widely adopted as the primary substrate material for high-precision, high-load, and geometrically complex cutting tools owing to its high hardness [2], excellent toughness [3], and outstanding red hardness (retaining a Vickers hardness above 55 HRC after exposure to 600 °C) [4]. However, under harsh conditions such as high-speed heavy cutting and dry cutting [5], the wear resistance and thermal stability of uncoated HSS tools often fail to meet the actual service requirements. Therefore, depositing hard and wear-resistant coatings such as aluminum titanium nitride (AlTiN) onto the tool surfaces to achieve surface strengthening has become an important path to improve tool performance and meet the demands of efficient processing [6].

It should be noted that the actual service life of coated tools is governed by the synergistic interplay between the coating and the substrate. In addition to the coating’s intrinsic mechanical properties and microstructure stability, the adhesion strength (L_C_) at the coating–substrate interface constitutes a critical performance parameter [7]. Furthermore, the specific characteristics of the substrate including micro-structural features [8] (e.g., relative density, carbide morphology and distribution) and mechanical properties [9] (e.g., Vickers hardness and bending strength σ) exert a significant influence on overall tool longevity. Insufficient interfacial bonding is prone to causing local coating peeling at the initial stage of cutting [7], while the higher hardness of the substrate helps to suppress the instability and plastic collapse of the coating under high contact stress [10], and excellent bending strength (σ) can delay the nucleation and propagation of cracks at the interface [11]. For PM M42 high-speed steel, the type, size, and spatial distribution of internal carbides are the core microscopic factors that regulate key performance indicators such as substrate hardness and bending strength (σ) [12].

Recent tribological research indicates that, apart from the field of cutting tools, the tribological integrity of surface layers and the interaction between nano-composite coatings and metal substrates have a decisive impact on the maintenance of functional characteristics and operational reliability of advanced mechanical components under extreme dynamic load conditions [13]. This understanding further highlights the importance of synergistically optimizing substrate materials and coating designs to achieve robust tribological performance.

During the preparation of PM M42 high-speed steel, the sintering temperature directly affects the densification degree of the substrate and determines the nucleation, growth, and distribution characteristics of primary carbides [14]. Subsequent heat treatment processes further influence hardness, bending strength, and the matching relationship between strength and toughness by regulating the phase transformation path and the precipitation behavior of secondary carbides [15]. Existing studies have shown that the coordinated optimization of sintering and heat treatment parameters can significantly improve the densification and uniformity of carbide distribution in the substrate, providing a reliable service performance basis for the design of high-performance coated tools [16]. However, existing research has predominantly focused on the composition design and deposition process optimization of AlTiN coatings [17], whereas systematic investigations into how substrate preparation methods and intrinsic substrate properties (microstructure, mechanical properties) influence the tribological performance of coated systems remain scarce.

Based on this, this study takes PM M42 HSS as the research object to systematically investigate the influence of different sintering densification (1130, 1160, and 1190 °C) and heat treatment (1150 °C for 0, 15, 60, and 120 min) processes on the microstructural evolution (e.g., densification, carbide phase transformation) and mechanical properties (e.g., hardness, bending strength) of the substrate. On this basis, a single-layer AlTiN coating is prepared by arc-PVD technology, and its friction and wear behavior (friction coefficient, wear rate) is quantitatively evaluated. By establishing a mechanism correlation framework of “substrate preparation process-substrate microstructure and mechanical properties-coating wear performance”, this study aims to provide theoretical references for the process optimization and coating design of PM M42/AlTiN coated cutting tool systems.

This work is particularly relevant to the journal Materials as it successfully bridges the two core issues of powder metallurgy high-speed steel substrate processing and coating tribology. Unlike previous studies that mainly focused on the regulation of AlTiN coating composition or the optimization of deposition parameters, this paper systematically correlates sintering and heat treatment processes with the microstructure evolution and mechanical properties of the substrate, and further extends to coating integrity and in-service wear behavior, thereby establishing an integrated analysis framework of “process-structure-performance-service performance” for powder metallurgy M42/AlTiN tool systems. This comprehensive perspective not only provides practical process guidance for industrial tool applications but also effectively expands the current understanding of the mechanism by which substrate regulation affects the tribological properties of coated steel systems.

## 2. Materials and Methods

### 2.1. Material Preparation

The experiment adopted the powder metallurgy process to prepare M42 high-speed steel. The raw material powder (purity above 99.9%, particle size: 1–5 μm) was provided by Nangong Fufan Metal Materials Co., Ltd. (Nangong, China), and its corresponding designed composition (wt.%) is shown in Table 1.

The raw material powder was mixed with 2 wt.% solid paraffin (forming agent) and wet ball-milled in a planetary ball mill (Miqi, Changsha Miqi Instrument Co., Ltd., Changsha, China) with anhydrous ethanol as the medium. The ball-milling process parameters were as follows: hard alloy grinding balls, ball-to-powder ratio of 8:1 (wt.%), rotational speed of 258 rpm, and ball-milling time of 16 h. The slurry after ball-milling was vacuum-dried, sieved (325 mesh), and then subjected to bidirectional pressing at a pressure of 600 MPa.

The compacted specimens were sintered in a vacuum sintering furnace (GSL-1750X tube furnace, manufactured by Hefei Kejing Materials Technology Co., Ltd., Hefei, China). The sintering process was as follows: heating at a rate of 5 °C/min to 350 °C and holding for 1 h to remove the binder, then heating at the same rate to 1130, 1160, and 1190 °C, respectively, and holding for 1 h, followed by cooling in the furnace to room temperature. The obtained samples were marked as S1, S2, and S3 according to different sintering temperatures. The sample information is shown in Table 2.

The sintered S3 sample (1190 °C) was selected for subsequent vacuum heat treatment. The heat treatment process was as follows: heating at a rate of 10 °C/min to 500 °C and holding for 30 min for preheating, then heating at a rate of 15 °C/min to 1150 °C, followed by austenitizing at 1150 °C for 15, 60, and 120 min, respectively. After that, the samples were oil-quenched to room temperature. Tempering was conducted immediately after oil quenching by heating at a rate of 3 °C/min to 540 °C and holding for 2 h, followed by furnace cooling. The resulting samples were marked as A1, A2, and A3 according to different austenitizing times. The sample information is shown in Table 3.

### 2.2. Coating Deposition

AlTiN coatings were deposited onto the surfaces of M42 high-speed steel substrates (S1–S3 and A1–A3) using arc plasma physical vapor deposition (arc-PVD) technology under a high-purity nitrogen atmosphere (99.999% N_2_). A composite Al-Ti target with an Al/Ti atomic ratio (at.%) of 30:70 was employed. The process parameters were as follows: deposition temperature was 550 °C, and bias voltage was −40 V. The as-deposited coating thickness was approximately 3 μm.

### 2.3. Organization Characterization and Performance Testing

#### 2.3.1. Microstructure and Mechanical Properties of the Substrate

The microstructure of the substrate was characterized by scanning electron microscopy (SEM, SU1510, Hitachi Ltd., Tokyo, Japan) equipped with energy-dispersive X-ray spectroscopy (EDS). Before characterization, the samples were wet-ground with SiC sandpapers of 400, 1000 and 2000 mesh successively and then polished and etched with a 4% nitric acid alcohol solution. The Vickers hardness (HV) of each sample (S1–S3 and A1–A3) was measured using a Q10A+ Vickers hardness tester (Qness GmbH, Golling, Austria) under a load of 500 g and a dwell time of 10 s. The average value of five valid points was taken for each group of samples. The bending strength (σ) of each sample was evaluated by the three-point bending method. The sample size was 5.25 mm × 6.5 mm × 30 mm, and the span was set to 14.5 mm. The bending strength (σ) was calculated according to the following formula.

(1) $$\sigma =\frac{3}{2}\frac{F_{\mathrm{bb}}L_{s}}{bh^{2}}$$

In the formula:

σ: Bending strength (MPa).

F_bb_: Maximum bending force (N).

h and b: Height and width of the sample (mm).

L_s_: The span between the two supports (mm); in this study, Ls = 14.5 mm.

#### 2.3.2. Microstructure and Mechanical Properties of the Coatings

The surface/cross-sectional microstructure, elemental distribution, and phase composition of the coatings were characterized by SEM-EDS and X-ray diffraction (XRD). The wear resistance of the coatings was evaluated on a Bruker (CETR) UMT-2 testing machine. The counter body was a 4 mm diameter Si_3_N_4_ ceramic ball. The test was conducted in reciprocating sliding mode under strictly controlled conditions to simulate the actual friction behavior and increase the objectivity of the test results. The test parameters were as follows: frequency = 5 Hz, amplitude = 2.5 mm, normal load = 5 N, and total cycles = 4500 (sliding time 900 s).

Due to the limited number of coated specimens available, each tribological test was performed once per condition. To provide an estimate of measurement reliability, the wear volume of each sample was determined by integrating multiple cross-sectional profiles acquired along the entire wear track using a non-contact optical profilometer (MicroXAM-800, KLA-Tencor Corp., Milpitas, CA, USA), and the wear rate (W_A_) was calculated accordingly. The calculation formula is as follows:

(2) $$W_{A}\;=\;\frac{V}{F\;\times \;L}$$

In the formula:

W_A_: Sliding wear rate (10^−6^ mm^3^·N^−1^·m^−1^).

V: Coating wear volume (mm^3^), obtained by multiplying the average cross-sectional area of the wear scar by the length of the wear scar.

F: Normal load (N).

L: Total sliding distance (m).

## 3. Results and Discussion

### 3.1. Influence of Sintering Temperature (1130, 1160, and 1190 °C) on the Microstructure and Properties of M42 High-Speed Steel

**Figure 1:**
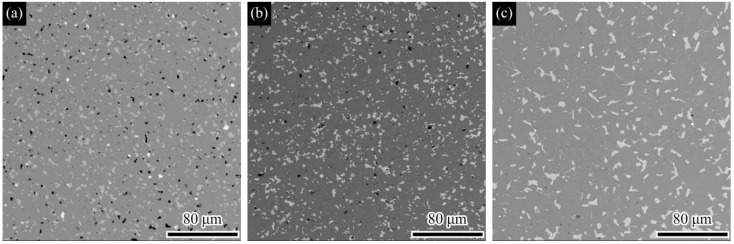
SEM micrographs showing the microstructure (pores and carbides) of M42 high-speed steel sintered at different temperatures: (**a**) 1130 °C; (**b**) 1160 °C; (**c**) 1190 °C.

The microstructures of the M42 high-speed steel samples sintered at different temperatures (1130 °C, S1; 1160 °C, S2; 1190 °C, S3) are shown in Figure 1. As the sintering temperature increases, the shrinkage and pore closure are enhanced, and the degree of material densification improves. At 1130 °C (S1), the sample exhibits typical under-sintered characteristics [18], with a large number of residual pores; when the temperature rises to 1160 °C (S2), the pore defects are reduced; and at 1190 °C (S3), it is difficult to identify obvious pores in the microstructure, indicating that the material has basically achieved high densification.

The results in Figure 1 also reveal that the carbides in M42 high-speed steel exhibit measurable coarsening with increasing sintering temperature, consistent with the morphological evolution expected from Ostwald ripening [19]. Specifically, at 1130 °C (S1), the carbides are mainly fine, relatively indistinct bright white particles, and are distributed diffusely. When the temperature rises to 1160 °C (S2), the particle boundaries become clearer, and they begin to grow and show local enrichment. When the temperature further increases to 1190 °C (S3), the carbides coarsen further, forming larger-sized, clearly defined blocky and short rod-like morphologies, with the local enrichment feature becoming more prominent.

**Figure 2:**
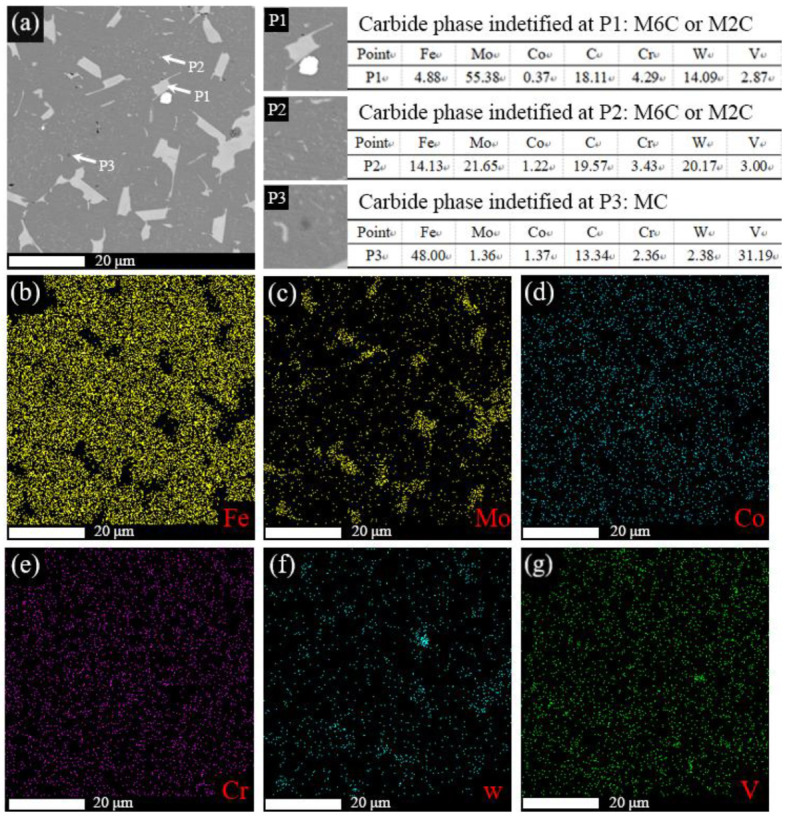
SEM and EDS analysis of M42 high-speed steel sintered at 1190 °C (S3): (**a**) SEM micrograph with EDS point analyses at P1, P2 and P3 (compositions are labeled adjacent to each point); (**b**) Fe mapping; (**c**) Mo mapping; (**d**) Co mapping; (**e**) Cr mapping; (**f**) W mapping; (**g**) V mapping.

M42 high-speed steel is a typical tungsten-molybdenum series cobalt-containing super-hard high-speed steel [20]. The strengthening effect of its as-cast microstructure mainly results from the precipitation and distribution of alloy carbides. In this study, the S3 sample sintered at 1190 °C was taken as the research object. The morphology, phase composition, and distribution of carbides within the matrix were systematically characterized by SEM-BSE imaging combined with EDS analysis, and the results are shown in Figure 2. The analysis indicates that there are mainly two types of primary carbides in the S3 sample:

(1) W/Mo-rich carbides: These carbides appear bright white in BSE images and exhibit morphologies primarily as irregular blocks or short rods; they constitute the dominant carbide phase in the sample. EDS analysis reveals enrichment of W and Mo at both P1 and P2 positions, while V content is relatively low, which is consistent with the compositional characteristics of M_2_C and/or M_6_C carbides [21].
(2) V-rich carbides: These carbides appear dark gray in BSE images and are predominantly distributed as isolated particles within the matrix. EDS analysis reveals that the V content at the P3 position is as high as 31.39 wt.%, which is consistent with the compositional characteristics of MC-type carbides and mainly exists in the form of VC [22].

Based on the raw material ratio used in this study (Table 1), at 1190 °C, the initial carbide precursors (WC, Mo_2_C, Cr_3_C_2_, VC) partially dissolve, accompanied by elemental diffusion. During the subsequent furnace cooling process, the supersaturated solute atoms re-precipitate to form composite carbides such as MC, M_2_C and M_6_C.

**Figure 3:**
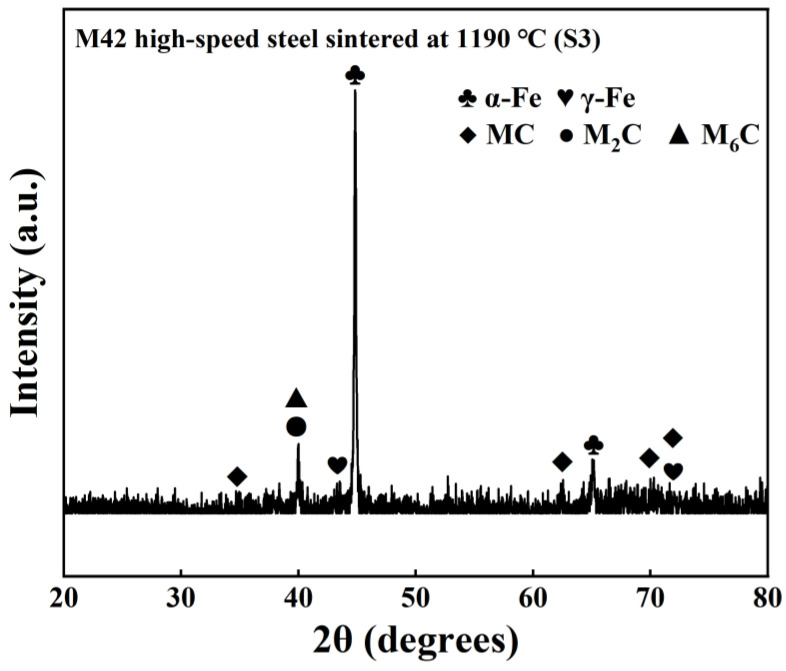
XRD pattern of M42 high-speed steel sintered at 1190 °C (S3).

Figure 3 shows the X-ray diffraction (XRD) pattern of sample S3 at room temperature. The results indicate that the phase composition of this sample is mainly composed of α-Fe ferrite, with a small amount of residual austenite (γ-Fe) and MC, M_2_C, and M_6_C carbides (M = Mo, Cr, V, etc.). No obvious diffraction peaks corresponding to carbide types, such as M_23_C_6_ or M_7_C_3_ phases, were detected. Furthermore, some characteristic peaks of M_2_C and M_6_C overlap, making it difficult to distinguish them precisely. The XRD analysis results are consistent with the SEM/EDS characterization results shown in Figure 2 and with those reported in the literature [23].

**Figure 4:**
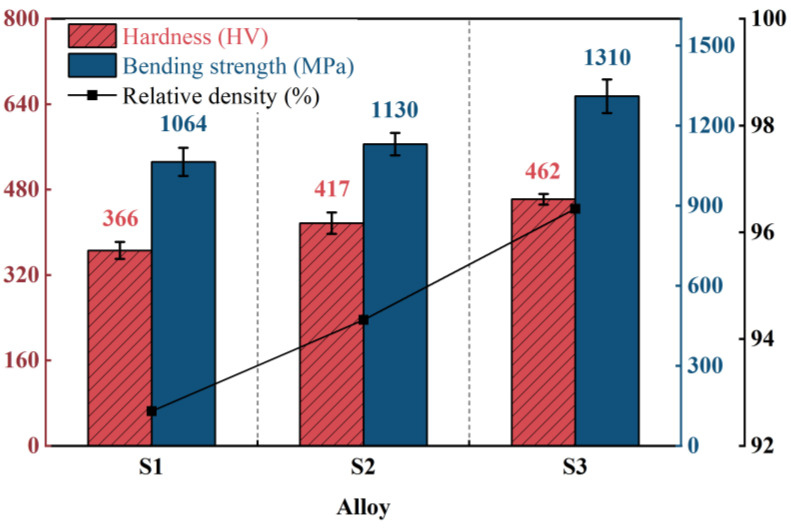
Vickers hardness, Bending strength (σ) and Relative density of M42 high-speed steel sintered at 1130 °C (S1), 1160 °C (S2), and 1190 °C (S3). Error bars represent the standard deviation (for hardness: *n* = 12 at 1130 °C, *n* = 18 at 1160 °C, *n* = 12 at 1190 °C; for bending strength: *n* = 2 per condition).

Figure 4 presents the Vickers hardness and bending strength (σ) of M42 high-speed steel sintered at three temperatures: 1130 °C (S1), 1160 °C (S2), and 1190 °C (S3). The results indicate that, within this range, both mechanical properties increase concurrently as the sintering temperature rises. The bending strength (σ) increases from 1064 ± 53 MPa (S1) to 1310 ± 63 MPa (S3), representing an improvement of approximately 23.8%. Correspondingly, the Vickers hardness rises from 366 ± 16 HV to 462 ± 10 HV, an increase of approximately 26%. The improvement in these properties is mainly attributed to the densification process promoted by the increase in sintering temperature (Figure 4), which effectively reduces stress concentration caused by pores and enhances the material’s load-bearing capacity and resistance to plastic deformation [24]. Based on the evaluation of the mechanical properties of each sample, the S3 sample, which has the best comprehensive mechanical properties and densification level, was selected as the benchmark sample for subsequent heat treatment process research. A well-densified substrate with elevated hardness is expected to provide improved mechanical support for the AlTiN coating, thereby enhancing the overall load-bearing capacity of the coating/substrate system during service.

### 3.2. Influence of Austenitizing Time on the Microstructure and Mechanical Properties of M42 High-Speed Steel

**Figure 5:**
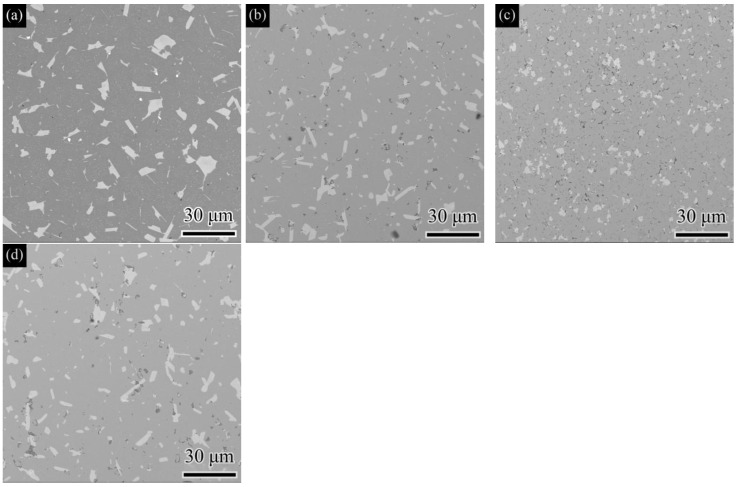
SEM micrographs showing the microstructure evolution of M42 high-speed steel austenitized for different times: (**a**) 0 min; (**b**) 15 min; (**c**) 60 min; (**d**) 120 min.

Taking the S3 sample as the baseline, austenitizing was carried out at 1150 °C for 0, 15, 60, and 120 min, respectively, followed by oil quenching and tempering as described in Section 2.1. The SEM morphologies of samples subjected to different austenitizing times (S3, A1, A2, and A3) are shown in Figure 5. The results indicate that, with increasing austenitizing time, the carbide morphology within the matrix exhibits staged evolution:

For the as-received sample (S3), the matrix contains large, irregular and well-defined bright white carbides (primarily M_2_C or M_6_C phases according to prior phase analysis). After austenitizing for 15 min (A1), the morphology and distribution of these carbides show little change, indicating that phase transformation kinetics under this condition are limited and that the process remains in the incubation period [25]. After austenitizing for 60 min (A2), the large carbides partially dissolve and spheroidize, transforming into fine, dispersed, nearly spherical particles. Meanwhile, a large number of fine secondary carbides (primarily MC phase) precipitate within the matrix and become locally enriched at the original large carbide/matrix interfaces and grain boundaries. This phenomenon may be related to the lower nucleation barrier at interfaces and grain boundaries and enhanced elemental diffusion kinetics [26]. After extending the austenitizing time to 120 min (A3), Ostwald ripening dominates carbide growth [19], and solute atoms diffuse through the matrix and accumulate in larger particles, resulting in further coarsening of the secondary carbides.

The above-mentioned microstructural evolution suggests a possible phase transformation mechanism involving M_2_C, M_6_C, and MC carbides during austenitizing at 1150 °C [27]. It is plausible that under thermal activation, the metastable M_2_C phase decomposes and releases solute atoms including W, Mo, V, and C. Among these, V, as a strong carbide-forming element, preferentially combines with C to nucleate and precipitate thermodynamically stable MC carbides (primarily VC) within the matrix; the remaining W, Mo, Fe, and C atoms then recombine via diffusion to precipitate a small amount of M_6_C carbides (e.g., Fe_3_(W, Mo)_3_C). The proposed phase transformation pathway is summarized as follows [28]:

(3) $$M_{2}C\;\to \;M_{6}C\;+\;\mathrm{MC}$$

Among them, the precipitation reaction of MC can be expressed as:

(4) V + C → VC

The precipitation reaction of M_6_C can be expressed as:

(5) $$3\mathrm{Fe}\;+\;3(W,\;\mathrm{Mo})\;+\;C\;\to \;{\mathrm{Fe}}_{3}{\left(W,\;\mathrm{Mo}\right)}_{3}C$$

During the 60 min austenitizing stage, the decomposition–reprecipitation process described above appeared most evident, characterized by local dissolution and spheroidization of the M_2_C phase and dispersed precipitation of the MC phase. Upon further extending the austenitizing time to 120 min (A3), the Ostwald ripening effect intensified. The residual trace M_2_C phase, due to its higher interfacial energy [27], tended to dissolve completely, whereas the M_6_C and MC carbides coarsened further, ultimately exhibiting the evolution characteristics shown in Figure 5.

**Figure 6:**
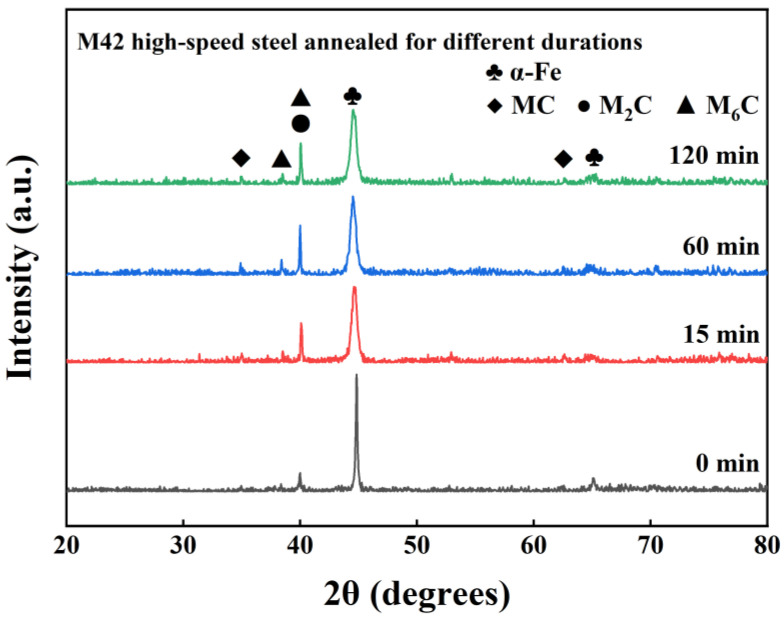
XRD patterns of M42 high-speed steel austenitized for different durations (0, 15, 60, and 120 min). The phase composition of each sample is consistent. Only the sample labeled 120 min is annotated to enhance clarity.

Figure 6 shows the X-ray diffraction (XRD) patterns of the samples (S3, A1, A2, and A3) subjected to different austenitizing times. The results indicate that although the SEM morphologies of the samples in different austenitizing conditions have evolved, the overall peak shapes and main peak positions in the XRD patterns have changed little, suggesting that the phase transformation of these carbides is primarily a continuous microstructural reconstruction rather than an abrupt phase transition.

**Figure 7:**
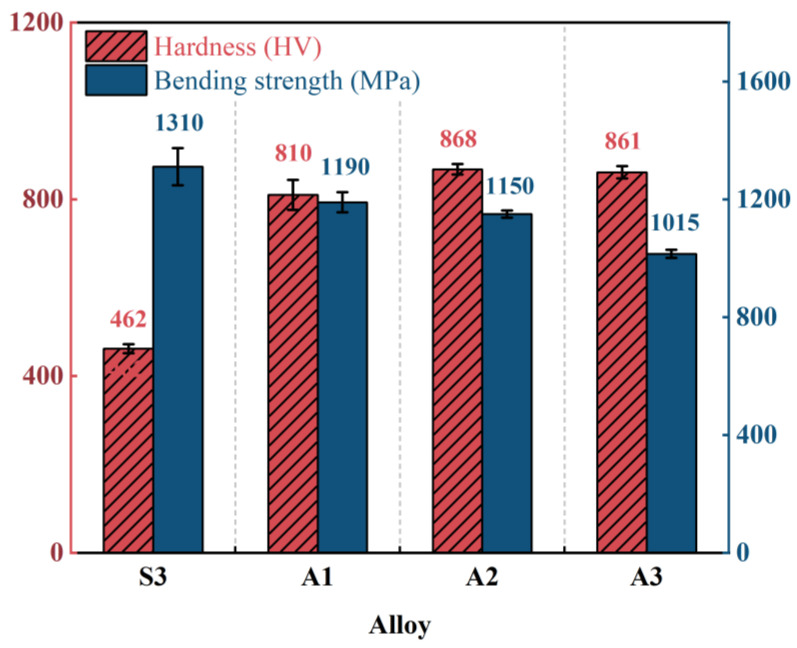
Hardness and Bending strength (σ) of M42 high-speed steel austenitized for different durations (0 min/S3, 15 min/A1, 60 min/A2, 120 min/A3). Error bars represent the standard deviation (for hardness: *n* = 12 for S3, *n* = 6 for A1, *n* = 9 for A2, *n* = 6 for A3; for bending strength: *n* = 2 per condition).

Figure 7 presents the Vickers hardness and bending strength (σ) of M42 high-speed steel austenitized for different durations (0 min/S3, 15 min/A1, 60 min/A2, 120 min/A3). The results show that hardness exhibits a non-monotonic trend, initially increasing and then slightly decreasing, reaching a peak value of 868 ± 12 HV at 60 min. In contrast, bending strength (σ) decreases monotonically from 1310 ± 63 MPa (S3) to 1015 ± 14 MPa (A3).

The above differences arise from the distinct responses of the two properties to microstructural evolution. The variation in hardness is mainly controlled by the precipitation strengthening effect [29], which stems from the decomposition of the metastable M_2_C phase and the precipitation of secondary carbides (primarily M_6_C and MC). This precipitation strengthening is most evident after 60 min of austenitizing (868 HV). Upon further extending the austenitizing time to 120 min, the secondary carbides gradually coarsen via Ostwald ripening, interface coherency decreases [30], and the precipitation strengthening effect weakens, resulting in a slight decrease in hardness.

In contrast, the bending strength (σ) is more sensitive to microstructural defects [31]. During the initial austenitizing stage (0–60 min), although the precipitation of secondary carbides increases the hardness, the increase in the number density of hard particles intensifies local stress concentration, which may induce crack initiation and propagation, leading to a decrease in bending strength. Further extending the austenitizing time (120 min) causes carbide coarsening and local enrichment, resulting in an increase in particle spacing and a reduction in the interfacial bonding strength between carbides and the matrix, thereby weakening crack propagation resistance [32] and further reducing bending strength. Based on the mechanical property evaluation, the choice of austenitizing time for M42 steel involves a trade-off between hardness and toughness: intermediate austenitizing conditions (60 min) are conducive to achieving a higher hardness level, while shortening the austenitizing time helps to mitigate the deterioration of bending strength.

Based on the mechanical property evaluation, the A2 sample (austenitized for 60 min) exhibits the highest hardness, which is expected to provide enhanced load-bearing support for the subsequently deposited AlTiN coating. However, the reduced bending strength may compromise the overall toughness of the coating/substrate system, a factor that will be evaluated in the subsequent tribological analysis.

### 3.3. Friction and Wear Behavior of AlTiN Coatings

Depositing a highly wear-resistant AlTiN coating on the surface of M42 high-speed steel is an effective technical approach to enhance tool performance and meet the demands of high-efficiency machining [6]. In the tribological performance evaluation, the friction coefficient and wear rate represent the interfacial shear response characteristics and the material removal behavior, respectively, which are two core indicators for assessing the wear resistance of the coating [33]. Based on this, this study systematically determined the friction coefficient and wear rate of the AlTiN coating under different substrate sintering temperatures (°C) and austenitizing times (min). By combining the three-dimensional morphology of the wear scar and the cross-sectional contour curve, the study revealed the evolution of its friction and wear behavior.

#### 3.3.1. Effect of Sintering Temperature

**Figure 8:**
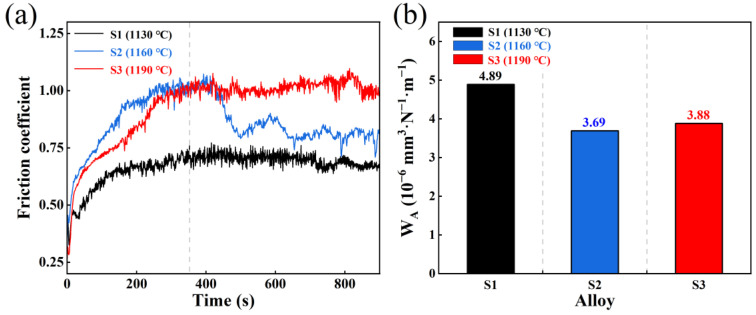
(**a**) Friction coefficient–time curves of AlTiN coatings on M42 high-speed steel sintered at different temperatures under dry friction conditions; (**b**) Wear rates of the corresponding samples.

The friction coefficient–time curves of AlTiN coatings under dry friction conditions at different substrate sintering temperatures (1130, 1160, and 1190 °C) are shown in Figure 8a. The results indicate that all three groups of samples experienced a brief running-in stage at the initial stage of friction, characterized by a rapid increase in the friction coefficient. The initial friction response during this stage was insensitive to the sintering temperature. As the friction process progressed, the system gradually entered a steady-state friction stage, at which point the effect of sintering temperature on the friction behavior became more evident: the steady-state friction coefficient of the AlTiN coating increased continuously with increasing sintering temperature.

Among these, the S2 sample (1160 °C) exhibited more pronounced periodic fluctuations during the steady-state friction stage, suggesting that its friction interface may have been in a state of dynamic reconstruction. During this process, the formation and migration of wear debris likely occurred frequently [34], leading to continuous changes in the interface shear conditions. In contrast, although sample S3 (1190 °C) exhibited the highest steady-state friction coefficient, its friction curve showed the smallest fluctuation amplitude, implying that high-temperature sintering (1190 °C) may have promoted more continuous shear deformation along the sliding direction at the interface, thereby suppressing intense oscillations in the friction response.

The wear rate results (Figure 8b) indicate that the material removal behavior of the AlTiN coating varies with substrate sintering temperature. Although the steady-state friction coefficient increases monotonically with increasing sintering temperature, the wear rate exhibits a non-monotonic trend: the S2 sample (1160 °C) exhibited the lowest wear rate (3.69 × 10^−6^ mm^3^·N^−1^·m^−1^), followed by S3 (1190 °C) at 3.88 × 10^−6^ mm^3^·N^−1^·m^−1^, and S1 (1130 °C) at 4.89 × 10^−6^ mm^3^·N^−1^·m^−1^. Combined with the analysis of the friction coefficient–time curve (Figure 8a), the friction interface of the S2 sample (1160 °C) appears to be in a state of dynamic reconstruction, and its material removal may be dominated by progressive ploughing and restricted plastic migration [35], thereby suppressing the deep penetration of abrasive particles and large-scale flake spalling. In contrast, the interface shearing process of the S3 sample (1190 °C) is more continuous, making it prone to forming deep penetration grooves and resulting in a higher wear rate than that of S2.

**Figure 9:**
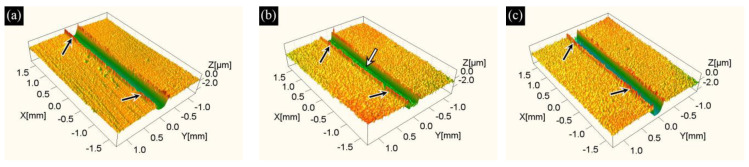
Three-dimensional wear scar morphologies of AlTiN coatings on M42 high-speed steel sintered at different temperatures: (**a**) 1130 °C/S1; (**b**) 1160 °C/S2; (**c**) 1190 °C/S3. Among them, the black arrows point to plastic flow, while the white arrows indicate ploughing features.

The three-dimensional wear scar morphology shown in Figure 9 reveals the material removal characteristics of the AlTiN coatings on samples sintered at different temperatures (S1, S2, S3) under dry friction conditions. The results indicate that the wear scar tracks of all samples along the sliding direction exhibit continuous groove structures, accompanied by varying degrees of plastic flow (black arrows in Figure 9) and ploughing features (white arrows in Figure 9).

The influence of substrate sintering temperature on the material removal behavior of the AlTiN coating system is reflected in the wear scar morphology (Figure 9). For the S1 sample sintered at 1130 °C, the wear groove exhibits a relatively straight contour and limited material accumulation on either side, indicating a relatively uniform material removal mode. Upon increasing the sintering temperature to 1160 °C (S2), the ploughing groove feature (white arrow in Figure 9) becomes more prominent, accompanied by discontinuous plastic flow accumulation, which suggests a higher degree of involvement of wear debris and results in increased spatial non-uniformity in material removal. When the sintering temperature is further increased to 1190 °C (S3), although the wear groove deepens further, its continuity along the sliding direction is notably improved, and the plastic flow deformation at the groove edge tends to stabilize, implying that the interface shearing and material migration processes become more continuous.

**Figure 10:**
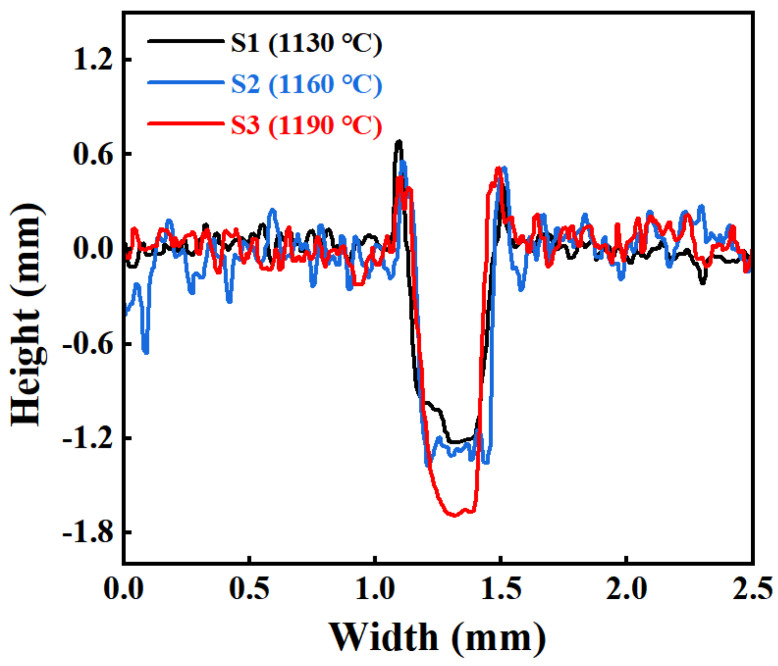
Cross-sectional profile curves of wear scars of AlTiN coatings on M42 high-speed steel sintered at different temperatures.

The cross-sectional profile curves (Figure 10) provide quantitative support for the above-mentioned differences in wear scar morphology. All three sample groups exhibit the typical “groove bottom–raised sides” profile characteristic, indicating that plastic flow of material extrusion along the groove edges to both sides occurs during the friction process. Comparative analysis of the cross-sectional profiles reveals that groove penetration depth generally increases with rising sintering temperature. Among these, the S3 sample (1190 °C) is characterized primarily by greater penetration depth, whereas the S2 sample (1160 °C) exhibits more pronounced profile undulations and spatial non-uniformity, suggesting enhanced discontinuity in the material removal process. Ultimately, the morphological characteristics of non-uniform intermittent removal under intermediate-temperature sintering (1160 °C) and deep penetration continuous removal under high-temperature sintering (1190 °C) are presented.

#### 3.3.2. Effect of Austenitizing Time

**Figure 11:**
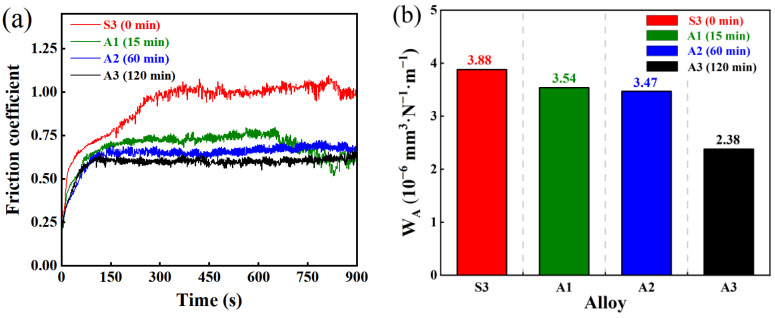
(**a**) Friction coefficient–time curves of AlTiN coatings on M42 high-speed steel austenitized for different durations under dry friction conditions; (**b**) Wear rates of the corresponding samples.

Under dry friction conditions, the friction coefficient–time curves of AlTiN coatings with different substrate austenitizing times (0, 15, 60, and 120 min) are shown in Figure 11a. The results indicate that all samples undergo a transition from the running-in stage to the steady-state friction stage during the initial stage of friction. This feature is also observed in samples with different sintering temperatures (Figure 8a), but their evolution behavior exhibits a dependence on the austenitizing time. As austenitizing time increases, the duration of the running-in period is shortened, suggesting that substrate austenitizing reduces the interface’s transient sensitivity to applied load and shear stress during the initial stage of friction. Combined with the redistribution and homogenization of residual stress during substrate austenitizing [36], extending the austenitizing time may suppress the interface stress fluctuations during the initial stage of friction and enhance the stability of the interfacial shear response [37], thereby accelerating the transition from the running-in stage to the steady-state friction stage.

After the system enters the steady-state friction stage, the regulatory effect of austenitizing time on the friction response becomes more evident. As austenitizing time increases, the steady-state friction coefficient exhibits an overall downward trend, and the fluctuation range of the friction coefficient decreases, indicating increased stability of the steady-state friction process. Compared with the as-received sample (S3), the 120 min austenitized sample (A3) not only exhibits the lowest steady-state friction coefficient but also shows improved smoothness and repeatability in the friction coefficient–time curve. The results suggest that sufficient substrate austenitizing improves load transfer and stress distribution at the coating–substrate interface [38], thereby enhancing the continuity and stability of the friction process and optimizing the steady-state friction performance of the system.

The variation in wear rate of the system under different austenitizing times is shown in Figure 11b, which is consistent with the evolution trend of the steady-state friction behavior. The results indicate that the wear rate decreases continuously with increasing substrate austenitizing time. The 120 min austenitized sample (A3) exhibits the lowest wear rate (2.38 × 10^−6^ mm^3^·N^−1^·m^−1^), which is approximately 39% lower than that of the as-received sample (S3). This suggests that full substrate austenitizing reduces coating volume loss and is expected to extend its service life [38].

It is worth noting that within the 15–60 min austenitizing range, although the substrate microstructure undergoes considerable evolution (Figure 5), the corresponding reduction in the coating’s wear rate remains relatively limited. This phenomenon indicates that the microstructural adjustment at this stage primarily reflects the evolution of the substrate itself, which is not sufficient to markedly enhance the overall load-bearing capacity of the coating–substrate system. However, when austenitizing time is extended to 120 min, the substrate microstructure and its mechanical response gradually achieve coordinated optimization [39], and both the load-bearing capacity of the coating–substrate interface and the stability of the wear process are enhanced, resulting in a clear decrease in wear rate.

**Figure 12:**
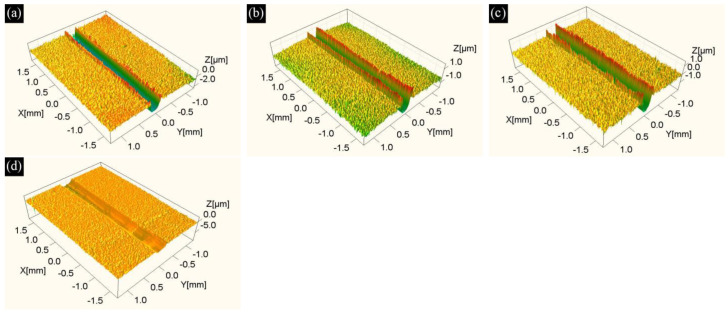
Three-dimensional wear scar morphologies of AlTiN coatings on M42 high-speed steel austenitized for different durations: (**a**) 0 min/S3; (**b**) 15 min/A1; (**c**) 60 min/A2; (**d**) 120 min/A3.

The three-dimensional wear scar morphologies shown in Figure 12 reveal the material removal characteristics of AlTiN coatings on samples with different substrate austenitizing times (S3, A1, A2, and A3) under dry friction conditions. Similar to the wear scar morphologies of samples with different substrate sintering temperatures (Figure 9), those of each austenitized sample exhibit continuous grooves along the sliding direction. Among these, varying degrees of material accumulation are observed on both sides of the wear grooves for samples S3, A1, and A2. In contrast, the wear scar surface of sample A3 is relatively flat overall, with limited material accumulation at the groove edges, indicating that the material removal process becomes more uniform and stable under extended austenitizing (120 min). In addition, no typical ploughing features attributable to abrasive particles are observed in the wear scars of samples S3–A3. Based on the characteristic morphologies observed under dry friction conditions, the material removal process appears to be predominantly governed by adhesive wear [40] and plastic flow.

**Figure 13:**
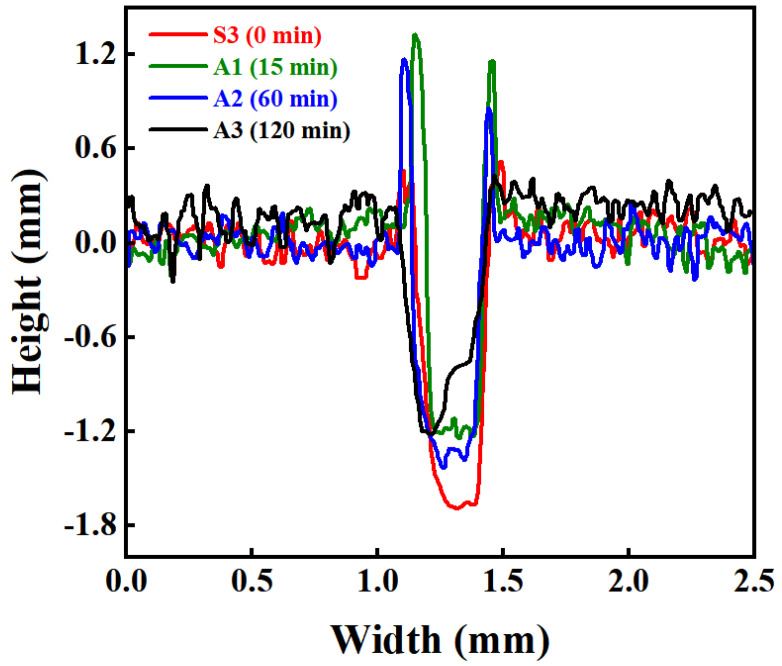
Cross-sectional profile curves of wear scars of AlTiN coatings on M42 high-speed steel austenitized for different durations.

The morphology of the wear scar (Figure 12) and the cross-sectional profile characteristics (Figure 13) of the AlTiN coating further reveal the influence of substrate austenitizing time on the coating’s material removal behavior. The results show that, under different austenitizing times, the degree of material accumulation on both sides of the wear grooves (A1 > A2 > S3 > A3) and the groove depth (S3 > A2 > A1 ≈ A3) exhibit a distinct non-monotonic variation trend. These two aspects reflect the differing response characteristics of plastic flow during lateral extrusion and normal cutting, respectively.

A closer examination of the mechanism suggests that material removal in samples S3-A3 may be predominantly governed by adhesive wear. Under this proposed mechanism, the friction load would be transmitted through micro-welded junctions on the coating surface [41], inducing local stress concentration. The location where this stress is released determines the spatial distribution and evolution path of plastic flow. Since the substrate’s plastic response influences the coating’s wear behavior, the evolution of the substrate’s microstructure with austenitizing time may alter the load transfer pathway at the coating–substrate interface [42], thereby affecting the shear stress distribution at the coating interface.

For the short-time austenitized sample (A1), the substrate microstructure undergoes only limited recovery, and the material retains a relatively high capacity for work hardening [43], which provides effective support for the coating and suppresses normal penetration of the counterbody into the wear scar, thereby reducing groove depth. Concurrently, shear stress concentrates along the groove edges, promoting increased material accumulation at these locations.

As the austenitizing time extends to 60 min, recovery processes progress and residual stresses are relieved [44], reducing the substrate’s work-hardening effect. The shear stress distribution appears to gradually shift from the groove edge toward the groove bottom, thereby likely reducing material accumulation while increasing groove depth.

When the austenitizing time is further extended to 120 min, the substrate’s microstructure approaches a more stable state and its work-hardening capacity decreases further. At this stage, the shear behavior in the micro-welding zone at the interface may transition from localized stress concentration to more dispersed deformation [42], and the distribution of shear stress tends to become more uniform with a reduction in local stress peaks. Plastic flow is mainly characterized by shallow repeated shearing and surface flattening, ultimately producing the morphological feature of the smallest groove depth and a relatively smooth wear scar surface.

#### 3.3.3. Wear Behavior in Relation to Substrate Properties

The friction-induced failure process of the AlTiN coating on the M42 high-speed steel substrate involves a dynamic coupling among coating loading, interface stress transfer, and substrate mechanical response [42]. Adjusting substrate characteristics, such as microstructure and mechanical properties, can alter the friction and wear behavior of the coating. The underlying mechanisms are related to the synergistic effects of load support, interface stress transfer, and microstructural evolution, as discussed below.

With respect to load support [45], the substrate hardness and bending strength (σ) jointly influence the elastic-plastic response of the AlTiN coating and substrate interface. Under reciprocating friction loading, a lower substrate hardness may promote local collapse and plastic instability in the substrate surface layer, intensifying the tensile and shear composite stress experienced by the coating. A lower σ is likely to induce local stress concentration, facilitating the initiation and propagation of interface microcracks and increasing the risk of coating spallation. It should be noted that the effects of hardness and σ on coating wear resistance do not simply superimpose linearly. Rather, they may exhibit synergistic or competitive relationships at different wear stages.

**Figure 14:**
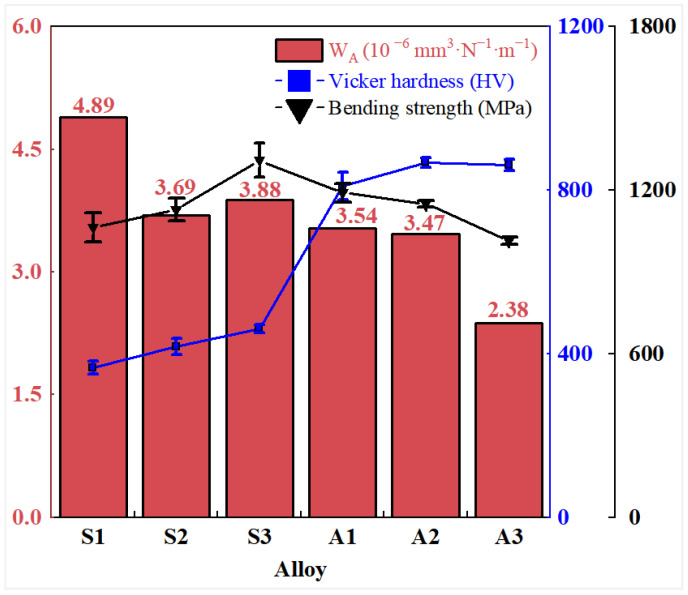
Experimentally measured Vickers hardness and bending strength (σ) of M42 high-speed steel, together with the wear rate (W_A_) of the corresponding AlTiN coating. Error bars for hardness and bending strength represent the standard deviation (for hardness: *n* = 12 for S1, *n* = 18 for S2, *n* = 12 for S3, *n* = 6 for A1, *n* = 9 for A2, *n* = 6 for A3; for bending strength: *n* = 2 per condition). Wear rate values were obtained from single tribological tests, as described in Section 2.3.2. Linear regression analysis of wear rate versus hardness is reported in the text (Section 3.3.3).

Figure 14 summarizes the experimentally measured hardness, bending strength (σ), and AlTiN coating wear rate (W_A_) for the six groups of M42 high-speed steel specimens, namely S1 through S3 and A1 through A3. The results show that W_A_ generally correlates negatively with hardness, whereas its relationship with σ is non-monotonic. This indicates that, under the present experimental conditions, increasing substrate hardness enhances the load-bearing capacity, reduces the level of tensile and shear composite stress and the accumulation of plastic strain at the interface [46], and helps to mitigate abrasive wear and spallation-type material removal. The influence of bending strength on coating wear behavior is primarily reflected in crack initiation, propagation, and the associated tendency for local spallation. Such effects may become more evident under higher loads or longer service durations [47].

A linear regression analysis of the wear rate versus substrate hardness was performed using the data from the six sample conditions (S1–S3 and A1–A3). The analysis yields the empirical relationship:

(6) $$W_{A}=5.254-0.00256\times HV$$

This corresponds to a Pearson correlation coefficient of r = −0.758 and a coefficient of determination R^2^ = 0.575. This negative correlation quantitatively supports the interpretation that increasing substrate hardness contributes to reduced coating wear. The moderate R^2^ value reflects the multifactorial nature of the wear process, wherein bending strength, interfacial adhesion, and carbide distribution also influence the wear behavior beyond hardness alone.

From the perspective of interface stress transfer, the coating–substrate adhesion strength (L_C_) characterizes the efficiency of tensile and shear stress transfer across the coating and substrate interface [48]. During reciprocating friction, contact stress initially accumulates within the coating and gradually transfers to the coating and substrate interface as the load increases. When the shear resistance and anti-debonding capacity of the interface are insufficient, local stress concentrations may develop at interfacial defects, promoting the formation and propagation of microcracks and ultimately leading to wear failure dominated by spallation [49]. A higher L_C_ value therefore generally corresponds to more stable interfacial stress transfer and a lower rate of interfacial damage [50], which can delay spallation failure. It should be noted that the influence of L_C_ on coating wear resistance exhibits stage-dependent characteristics. In the early stage of wear, it mainly acts to suppress the initiation of interfacial debonding [48]. In the middle and later stages, it primarily retards the spallation process by impeding crack propagation across the interface [48].

**Figure 15:**
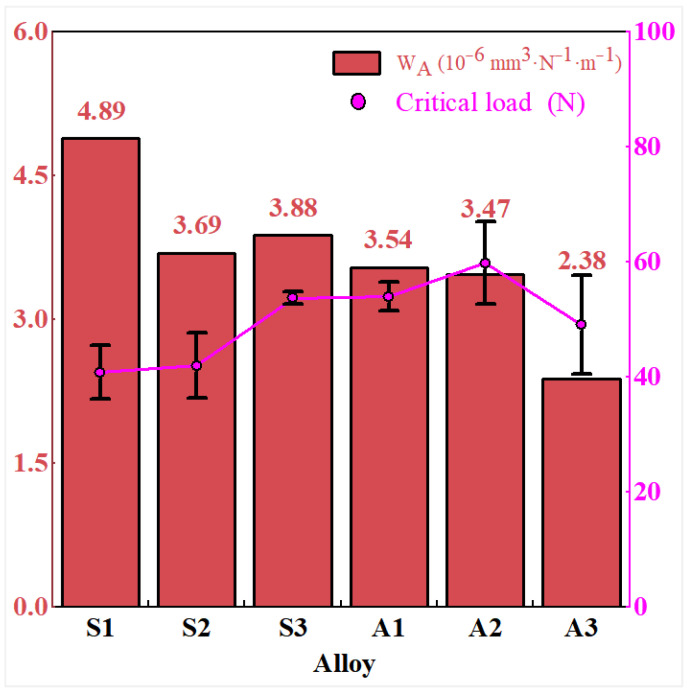
Experimentally measured wear rate (W_A_) of the AlTiN coating and critical load (L_C_) at the coating and substrate interface for samples S1 through S3 and A1 through A3. Error bars for L_C_ represent the standard deviation (*n* = 3 per condition). Wear rate values were obtained from single tribological tests.

To evaluate this aspect, the coating substrate adhesion strength (L_C_) was measured for the AlTiN coatings on samples S1 through S3 and A1 through A3 using the scratch method. A Rockwell C diamond indenter with a cone angle of 120 degrees and a tip radius of 0.2 mm was employed. A linearly increasing load from 0 to 150 N was applied over a scratch length of 6 mm. The critical load L_C_ was defined as the load at which the acoustic emission signal exhibited an abrupt change accompanied by visible coating spallation. Each sample was tested three times, and the average value is reported. The results are presented in Figure 15.

The results in Figure 15 indicate an overall negative correlation between W_A_ and L_C_. As L_C_ increases from approximately 40 N to about 60 N, W_A_ tends to decrease, suggesting that enhanced coating–substrate adhesion can help to reduce material removal. However, sample A3 exhibits a local deviation from this trend. Its L_C_ value is lower than that of sample A2, yet its wear rate is clearly reduced. When considered together with the hardness data and the three-dimensional wear scar morphology of sample A3 shown in Figure 12, it appears that the higher substrate hardness of A3 contributes to a shallower cutting depth and reduced plastic ploughing. This may partially offset the adverse effect of the lower L_C_ value.

With regard to microstructural evolution, the carbide phase transformation from M_2_C to M_6_C and MC is a key microstructural process that influences the wear resistance of the AlTiN coating. This influence likely operates through the pathway of carbide type and distribution, near-surface mechanical response of the substrate, and coating wear behavior [51]. The strengthening effects of different carbide types in the M42 high-speed steel matrix differ substantially. M_2_C is a typical eutectic carbide in M42 high-speed steel. Although it possesses relatively high intrinsic hardness, its coarse eutectic network morphology tends to facilitate crack initiation and propagation, thereby reducing material toughness. Its main contribution to strengthening arises from its high-temperature decomposition, which leads to the precipitation of fine, dispersed secondary carbides such as M_6_C and MC, producing a precipitation strengthening effect. M_6_C and MC exhibit good thermal stability and high hardness [52] and function as stable hardening phases that effectively pin dislocations and inhibit grain boundary migration, thereby enhancing both the hardness and the high-temperature load-bearing capacity of the material.

**Figure 16:**
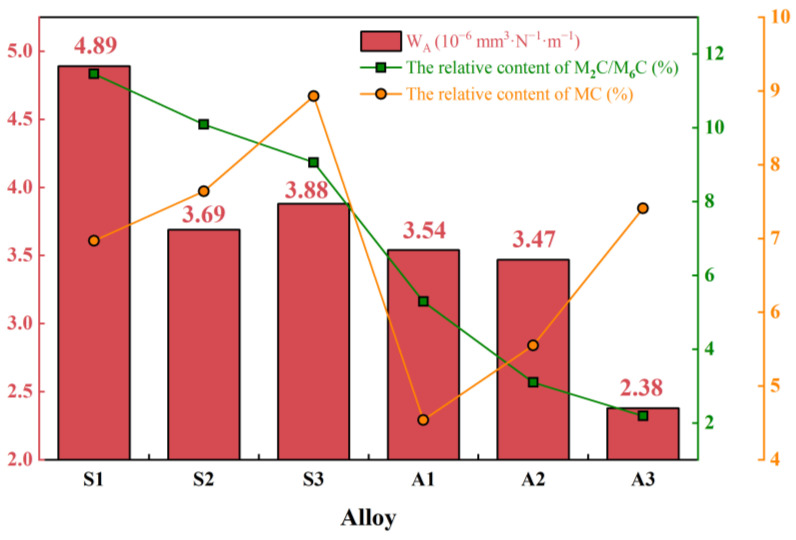
Experimentally measured wear rate (W_A_) of the AlTiN coating, together with the relative contents of the M_2_C and M_6_C composite phases and the MC phase in the substrate. Wear rate values were obtained from single tribological tests. Carbide content values were determined by grayscale threshold analysis of SEM-BSE images.

Based on these considerations, the relative contents of the various carbides in samples S1 through S3 and A1 through A3 were quantitatively analyzed using the grayscale threshold segmentation method with Image Pro Plus software (version 6.0). The results are shown in Figure 16. Because M_2_C and M_6_C exhibit similar morphological features in SEM BSE images, as shown in Figure 2 and Figure 5, and are difficult to distinguish reliably, they were grouped together as a composite M_2_C and M_6_C phase for statistical purposes.

The results in Figure 16 show that with increasing sintering temperature from S1 to S3 and extended austenitizing time from A1 to A3, the relative content of MC increases monotonically, whereas that of the M_2_C and M_6_C composite phases decreases continuously. This inverse relationship is consistent with a progressive transformation from M_2_C to M_6_C and MC. Under the combined effects of precipitation strengthening and the intrinsic strengthening contributions of MC and M_6_C, the substrate hardness increases steadily, as shown in Figure 14, which in turn contributes to a reduction in the wear rate of the AlTiN coating. It should be noted that during the initial stage of austenitizing, corresponding to sample A1, the phase transformation is in the incubation period, as shown in Figure 5, and the microstructural evolution exhibits non-equilibrium characteristics. The original M_2_C phase partially dissolves, while the M_6_C and MC phases have not yet nucleated or grown effectively, leading to a noticeable decrease in the relative contents of both the M_2_C and M_6_C composite phases and the MC phase at this stage.

In summary, the wear behavior of the AlTiN coating is influenced by a combination of substrate hardness, bending strength (σ), coating substrate adhesion strength (L_C_), and carbide phase evolution from M_2_C to M_6_C and MC. Among these factors, the carbide phase evolution appears to not only govern the levels of substrate hardness and bending strength but also indirectly affect the coating–substrate adhesion strength by altering carbide distribution uniformity and the interfacial stress state. Ultimately, this microstructural evolution appears to play a dominant role in determining the coating wear behavior under reciprocating friction. A comprehensive comparison of the six sample groups, S1 through S3 and A1 through A3, indicates that with the minimization of wear rate as the primary objective, the A3 condition, which corresponds to a sintering temperature of 1190 °C and an austenitizing time of 120 min, exhibited the lowest wear rate among the six sample groups evaluated in this study. This condition achieves a favorable combination of substrate load-bearing capacity, interfacial load transfer, and microstructural stability.

While the present study demonstrates that substrate heat treatment can effectively tailor the wear performance of AlTiN coatings through microstructural and mechanical optimization, further enhancements may be achievable through alternative strategies. For instance, the incorporation of nanoscale additives or surface modifications has been shown to markedly influence the tribological characteristics of advanced coating systems [13]. Such approaches could complement the substrate engineering methodology established herein and represent a promising direction for future work.

## 4. Conclusions

This study employed powder metallurgy M42 high-speed steel (PM M42 high-speed steel) as the substrate and systematically investigated the influence of sintering temperature (1130, 1160, and 1190 °C) and austenitizing time (1150 °C for 0, 15, 60, and 120 min) on the microstructure and mechanical properties (hardness, bending strength) of the substrate. An AlTiN hard coating was deposited onto the substrate surface via arc-PVD to further elucidate how substrate characteristics influence the tribological behavior (friction coefficient, wear rate) of the coating. The main conclusions are as follows:

(1) Increasing the sintering temperature from 1130 °C to 1190 °C promotes progressive densification of the M42 high-speed steel. The Vickers hardness increases from 366 ± 16 HV (S1) to 462 ± 10 HV (S3), and the bending strength rises from 1064 ± 53 MPa (S1) to 1310 ± 63 MPa (S3). The improved densification effectively reduces stress concentrations associated with residual porosity, thereby providing a mechanically superior substrate for the AlTiN coating.
(2) During austenitizing at 1150 °C, microstructural evolution consistent with a progressive carbide transformation from M_2_C to M_6_C and MC is observed. With increasing austenitizing time, the substrate hardness initially increases and then decreases slightly, reaching a peak value of 868 ± 12 HV after 60 min (A2). In contrast, the bending strength (σ) decreases monotonically, reaching 1015 ± 14 MPa after 120 min (A3), illustrating a trade-off between hardness and toughness.
(3) The wear behavior of the AlTiN coating is governed by the combined effects of substrate hardness, bending strength (σ), coating–substrate adhesion strength (L_C_), and carbide phase evolution. Elevated substrate hardness enhances the load-bearing capacity and reduces wear, whereas bending strength influences crack propagation and spallation tendency. The adhesion strength L_C_ governs the efficiency of interfacial stress transfer. The carbide phase evolution appears to modulate the wear response by dictating the substrate’s microstructural state and mechanical property matching.
(4) Among the six sample groups evaluated, the A3 condition (sintering at 1190 °C and austenitizing for 120 min) yields the lowest wear rate of 2.38 × 10^−6^ mm^3^·N^−1^·m^−1^, representing a 39% reduction relative to the as-sintered S3 baseline. This result is strictly valid under the specific conditions investigated in this work; further studies employing a full factorial design are required to evaluate potential interactive effects between sintering temperature and austenitizing time.
(5) The experimental findings confirm the initial hypothesis that substrate densification and austenitizing-induced carbide refinement improve the mechanical support for the AlTiN coating, thereby enhancing wear resistance. The observed non-monotonic trends in hardness and the deviation in the Lc–wear rate relationship for sample A3, however, were not fully anticipated and underscore the complex interplay among substrate microstructure, interfacial adhesion, and coating tribological response.

The optimized substrate condition identified in this study provides a promising route for fabricating M42 high-speed steel cutting tools with extended service life. These findings are directly relevant to high-performance machining applications in the automotive and aerospace industries, where improved tool durability under demanding conditions is of critical importance.

## Figures and Tables

**Table 1:** The designed composition of M42 high-speed steel (wt.%).

Alloy	Design Composition/wt.%					
	WC	Mo_2_C	Cr_3_C_2_	VC	Co	Fe
M42	1.60	10.10	4.39	1.48	8.50	73.93

**Table 2:** Sintered M42 high-speed steel samples corresponding to sintering temperatures of 1130, 1160, and 1190 °C.

Sample	M42 High-Speed Steel		
ST/°C	1130	1160	1190
No.	S1	S2	S3

ST: Sintering Temperature (°C).

**Table 3:** Heat-treated M42 high-speed steel samples corresponding to austenitizing times of 15, 60, and 120 min.

Sample	S3		
AT/min	15	60	120
No.	A1	A2	A3

AT: Austenitizing Time (min).

## Data Availability

The original contributions presented in this study are included in the article. Further inquiries can be directed to the corresponding authors.
